# Editorial: Circadian Rhythm: From Microbes to Hosts

**DOI:** 10.3389/fcimb.2020.613181

**Published:** 2020-11-02

**Authors:** Marina Maria Bellet, Kristin Eckel-Mahan, Luigina Romani

**Affiliations:** ^1^ Department of Experimental Medicine, University of Perugia, Perugia, Italy; ^2^ Institute of Molecular Medicine, McGovern Medical School, University of Texas Health Science Center at Houston, Houston, TX, United States

**Keywords:** circadian rhythm, bacteria, fungi, parasite, immune system, epigenetic, infections

Circadian rhythms govern almost every aspect of life on earth. From microbes to hosts, organisms from different domains of life are enabled to anticipate environmental changes, thanks to the presence of a molecular clock ticking in each cell. For several decades the circadian field has been focusing mainly on dissection of the core clock mechanism driving these 24-hour rhythms, thus reaching accepted models, conserved but distinct, in cyanobacteria, plants, fungi and mammals (Bell-Pedersen et al., 2005; Takahashi, 2017). Studies of circadian rhythms in alternate species are fewer to come by. Today, the circadian outputs, that is the physiological pathways controlled in an oscillatory fashion, have evoked increased interest. Their study is bringing us closer to understanding the adaptive meaning of the circadian rhythms over time, and the consequences for each species’ pathophysiology.

Circadian regulation of host immune responses to infection in mammals has been extensively documented in the last two decades of studies. Instead, how the circadian clock plays a role in controlling the physiology of microbial species, either commensal or pathogenic, and how such regulation can affect host-microbe interactions is still poorly understood.

The present research topic brings together seven articles summarizing the main knowledge pertaining to the circadian regulation of response to infections in mammals, the potential relevance of circadian rhythms in human pathogens, and the interactions between our body clock and that of commensal or pathogenic microrganisms.

In their review article, Costantini et al. provide an overview on the current knowledge related to the presence of circadian rhythms in pathogenic bacteria and fungi, and discuss the possible interactions between these microbes and their hosts and as a function of their own molecular clock. They also describe the profound influence they could have in each other’s behavioral and metabolic circadian activities. Finally, the article speculates on the potentially susbstantial impact of circadian medicine in infectious diseases. Carvalho Cabral et al. extends these concepts to parasites and parasitic infections. In their review, the authors highlight on different levels the interactions between host and parasite rhythms, including the presence of intrinsic, host-driven or vector-driven daily rhythms in protozoan or helmintic parasites, and changes in the host’s circadian rhythm induced by parasites. *Tripanosoma brucei* (*T. brucei*) parasitic infection is perhaps the most emblematic infection capable of disrupting the host’s circadian rhythm. The resulting disease, the “sleeping sickness”, as anticipated by its name, causes a potentially fatal disease characterized by sleep and circadian disruption. As described by Rijo-Ferreira and Takahashi, parasite invasion of the brain and neuroinflammation are likely responsible for this deregulated sleep. Moreover, the molecular clock of *T. brucei* is discussed, including how it controls many aspect of parasite metabolism, including its own susceptibility to antiparasitic drugs. How parasite and other microbes’ rhythm dictate changes in their hosts, and what the evolutionary advantage may hold for microbial species in this process still remain elusive.

In their mini review, Murakami and Tognini summarize the results of a series of works that have described the reciprocal circadian regulation between hosts and the commensal microbial population constituting the gut microbiota. The authors cover the role of the host circadian clock in dictating the structure and composition of the gut microbiota and the production of microbial metabolites, which in turn, are recognized by the innate immune system through which they signal to intestinal epitelial cells, by changing their circadian transcriptional program. Intriguingly, this is likely to occur through (among other mechanisms) epigenetic mechanisms, as suggested by Orozco-Solis and Aguilar-Arnal. The circadian regulation mostly relies on epigenetic modifications of chromatin accessibility (Sassone-Corsi, 2016), and the authors describe common epigenetic mechamisms shared by the circadian and immune system that might provide reciprocal interaction between the two systems.

Almost every aspect of the immune response in mammals is tightly regulated by the circadian clock, including immune cells abundance, trafficking, differentiation and cytokines production (Scheiermann et al., 2018), but also the activity of soluble mediators such as the complement system, for which there are evidence which suggest its dysregulation during circadian disruption, as summarized in the review by Shivshankar et al. The rhythmicity of immune functions, both at the steady-state and during infections, clearly affects host immune response to pathogens, as revealed in the last two decades through the use of many different murine models of infection. Even one of the most fearful evolutions of bacterial infections, which is sepsis, is under circadian regulation. Mul Fedele et al. in their article further characterize the diurnal response to septic shock, and clearly highlight the central role of TNFα signaling in this circadian response.

Overall, this Research Topic brings together a collection of articles which are meant to provide readers with an overview of our current understanding regarding the circadian regulation of microbial physiology and host immune responses to infections by these microbes. Collectively, they highlight the importance of circadian rhythms in the host-pathogen reciprocal interactions. The application of a circadian medicine in the field of infections, including viral infections, might represet the direct conceptual advance in this field, in which promising developments are expected in the near future (Greco and Sassone-Corsi, 2020), as evidenced by the urgent needs imposed by the pandemic spread of SARS-CoV-2 infection (Ray and Reddy, 2020).

The editors of this topic greatly appreciate the contributions to this collection given by all of the authors and reviewers.

## Author Contributions

MB, KE-M, and LR edited the research topic, contributed to the article and approved the submitted version.

## Funding

KE-M is funded by NIH grant DK114037.

## Conflict of Interest

The authors declare that the research was conducted in the absence of any commercial or financial relationships that could be construed as a potential conflict of interest.

## References

[B1] Bell-Pedersen D. Cassone V. M. Earnest D. J. Golden S. S. Hardin P. E. Thomas T. L. (2005). Circadian rhythms from multiple oscillators: lessons from diverse organisms. Nat. Rev. Genet. 6 (7), 544–556. 10.1038/nrg1633 15951747 PMC2735866

[B2] Greco C. M. Sassone-Corsi P. (2020). Personalized medicine and circadian rhythms: Opportunities for modern society. J. Exp. Med. 217 (6), e20200702. 10.1084/jem.20200702 32433754 PMC7971139

[B3] Ray S. Reddy A. B. (2020). COVID-19 management in light of the circadian clock. Nat. Rev. Mol. Cell Biol. 21 (9), 494–495. 10.1038/s41580-020-0275-3 32699357 PMC7374068

[B4] Sassone-Corsi P. (2016). “The Epigenetic and Metabolic Language of the Circadian Clock,” in A Time for Metabolism and Hormones. Eds. Sassone-Corsi P. Christen Y. (Cham (CH: Springer), 1–11. 28892346

[B5] Scheiermann C. Gibbs J. Ince L. Loudon A. (2018). Clocking in to immunity. Nat. Rev. Immunol. 18 (7), 423–437. 10.1038/s41577-018-0008-4 29662121

[B6] Takahashi J. S. (2017). Transcriptional architecture of the mammalian circadian clock. Nat. Rev. Genet. 18 (3), 164–179. 10.1038/nrg.2016.150 27990019 PMC5501165

